# A Case Study of Stevens–Johnson Syndrome-Toxic Epidermal Necrolysis (SJS-TEN) Overlap in *Mycoplasma pneumoniae*-Associated Tracheobronchitis

**DOI:** 10.1155/2019/5471765

**Published:** 2019-05-30

**Authors:** Ranjit Sah, Samikshya Neupane, Shusila Khadka, Sagar Poudyal, Hem Raj Paneru, Ranjana Sah, Sanjit Sah, Vivek Pant

**Affiliations:** ^1^Department of Microbiology, Thribuvan University Teaching Hospital, Institute of Medicine, Kathmandu, Nepal; ^2^Department of Medicine (Division of Infectious Disease), Medanta-The Medicity, Gurugoan, Haryana, India; ^3^Department of Pathology, Thribuvan University Teaching Hospital, Institute of Medicine, Kathmandu, Nepal; ^4^Institute of Medicine, Tribhuvan University, Kathmandu, Nepal; ^5^Department of Anesthesia (Critical Care), Thribuvan University Teaching Hospital, Institute of Medicine, Kathmandu, Nepal

## Abstract

Stevens–Johnson syndrome is a medical emergency which is characterized by skin and mucosal reaction to the use of certain drugs. Atypical Steven–Johnson syndrome can occur due to various microorganisms and *Mycoplasma pneumoniae* being one of them. We present a clinical course, diagnosis, and successful management of Steven–Johnson syndrome-toxic epidermal necrolysis (SJS-TEN) overlap due to *Mycoplasma pneumoniae* in a 17-year-old Nepalese female. In the resource-limiting country and hospitals where serology and PCR for *M. pneumoniae* is not easily accessible, a simple bedside cold agglutination test can be done to increase the suspicion of infectious cause (most common *M. pneumoniae* ) of SJS-TEN overlap. *M. pneumoniae* infection should be considered in all cases of mucositis, especially in patients having preceding respiratory tract infections (tracheobronchitis).

## 1. Introduction

Stevens–Johnson syndrome (SJS) is an immune-mediated disease characterized by a prodromal illness followed by severe mucocutaneous symptoms [1]. SJS and its more severe form, toxic epidermal necrolysis (TEN), are the result of an inflammatory response that results in keratinocyte necrosis and perivascular lymphocyte infiltration. Stevens–Johnson syndrome-toxic epidermal necrolysis (SJS–TEN) overlap has the characteristics of both SJS and TEN with the involvement of 10%–30% of the body surface area, epidermal detachment, fever, and malaise [1, 2]. SJS was classically related to a medication hypersensitivity reaction; however, infectious etiologies are increasingly recognized as inciting agents. The common pathogens are *Mycoplasma pneumoniae*, Enterovirus, hepatitis B virus, *Yersinia enterocolitica*, Epstein–Barr virus, group A streptococcus, and *Mycobacterium tuberculosis* [2]. *Mycoplasma pneumoniae* is a common cause of community-acquired pneumonia in all age groups. This pleomorphic bacterium lacks cell wall and attaches directly to respiratory epithelium, causing damage. Extrapulmonary manifestation in skin and mucosa due to this bacterium occurs in 25% of cases [3].

Infectious origin of SJS is suspected if infectious symptoms precede the onset of skin or mucosal lesion and serological diagnosis for suspected organism is positive [4]. Constitutional symptoms appear at an early stage followed by mucocutaneous involvement. Mucosal lesion is more common than cutaneous lesion and is more commonly seen in oral, genital, and ocular mucosal surfaces [2]. *Mycoplasma pneumoniae*-associated mucositis occurs through direct cytotoxic damage and through cross reacting auto antibody formation [5]. The histopathological diagnosis of SJS is demonstration of epidermal necrosis [6].

The purpose of reporting this case report is to increase awareness among clinicians about *Mycoplasma pneumoniae*-SJS relationship. To the best of our knowledge, this is the first case of *Mycoplasma pneumoniae*-associated SJS-TEN overlap reported from Nepal.

## 2. Case Presentation

**Figure 1:**
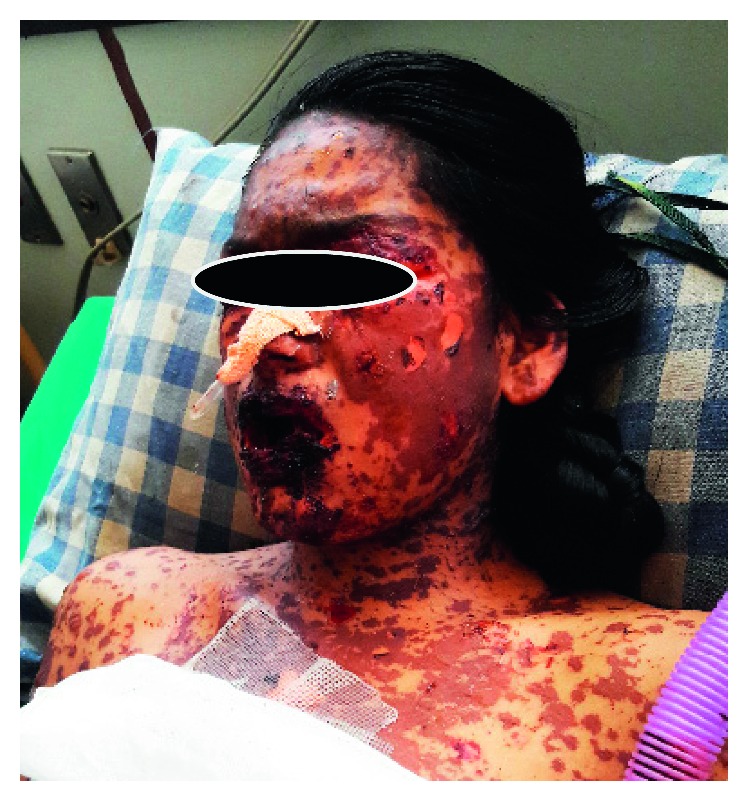
Skin rash over face and neck along with extensive blistering lesions with mucosal involvement.

**Figure 2:**
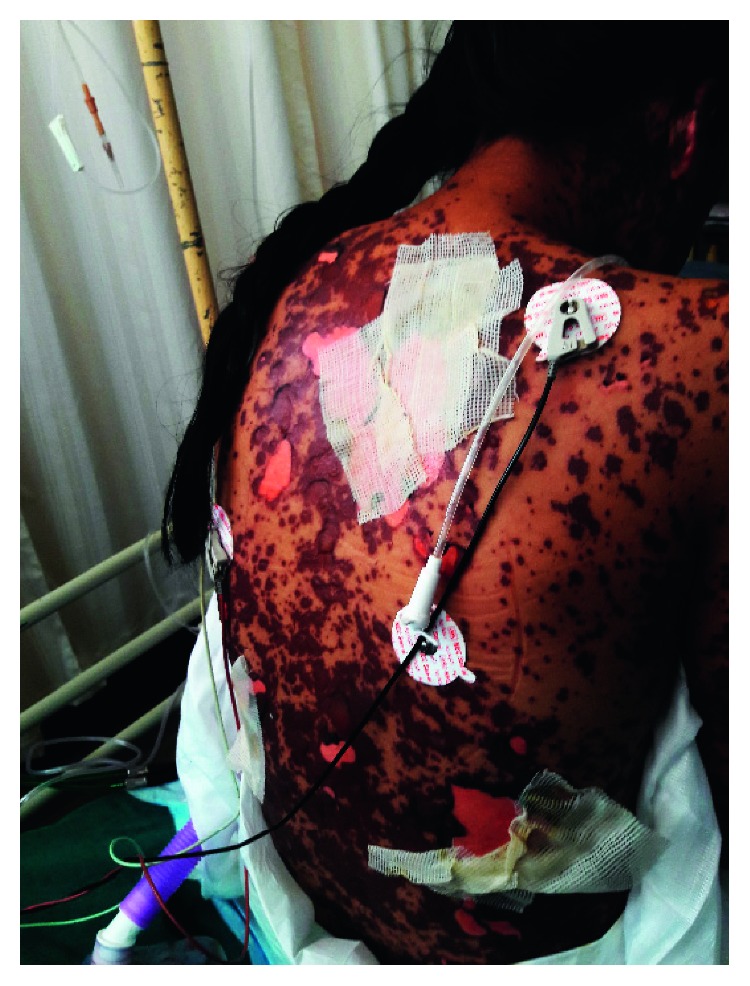
Skin rash over the back of trunk.

**Figure 3:**
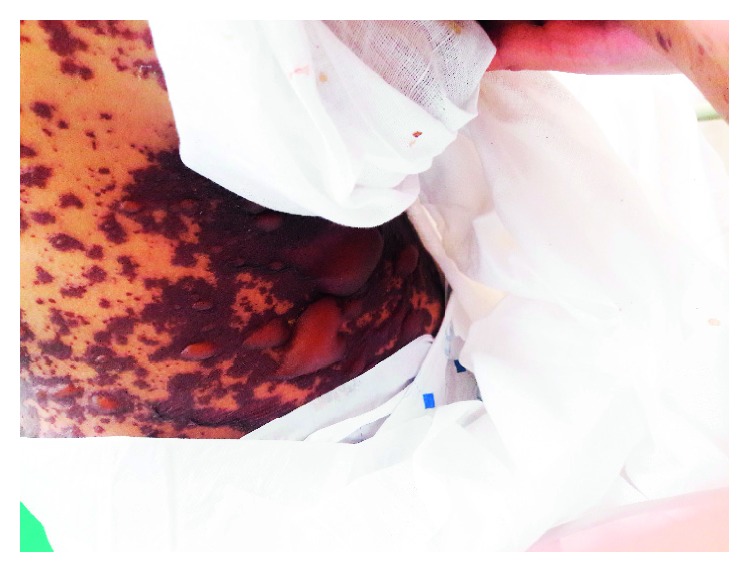
Skin rash with bullae formation.

**Figure 4:**
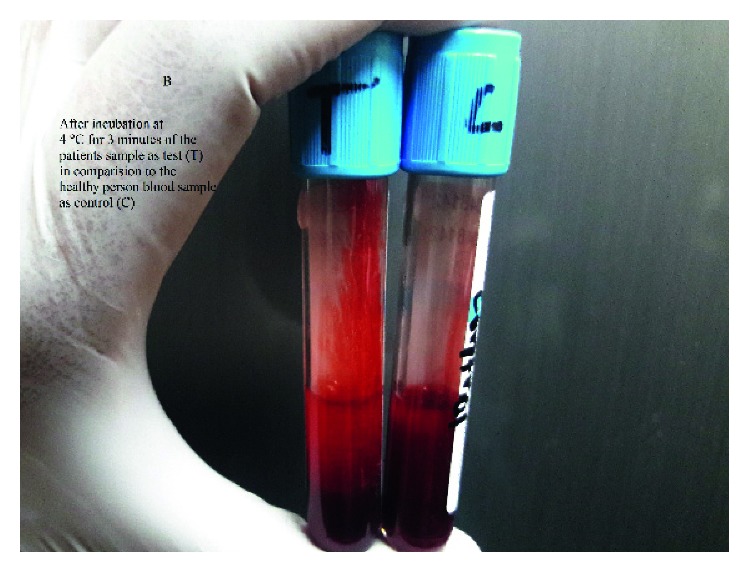
Clumping of red blood cell along the surface of the tube at 4°C.

**Figure 5:**
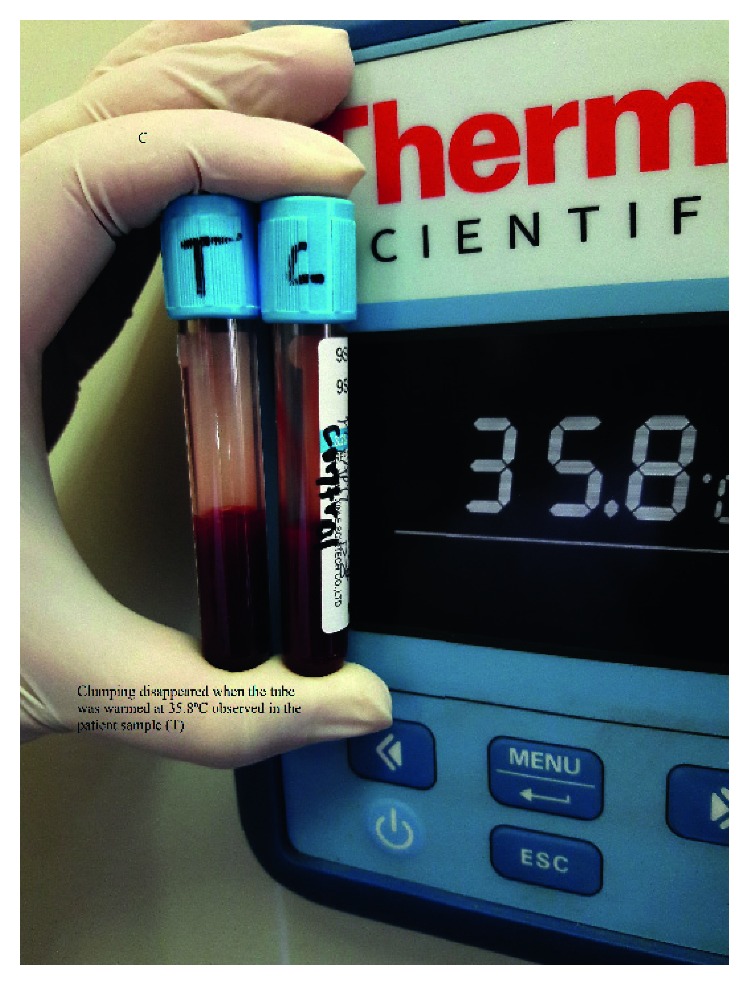
Disappearance of clumping while heating the tube at 36°C.

**Figure 6:**
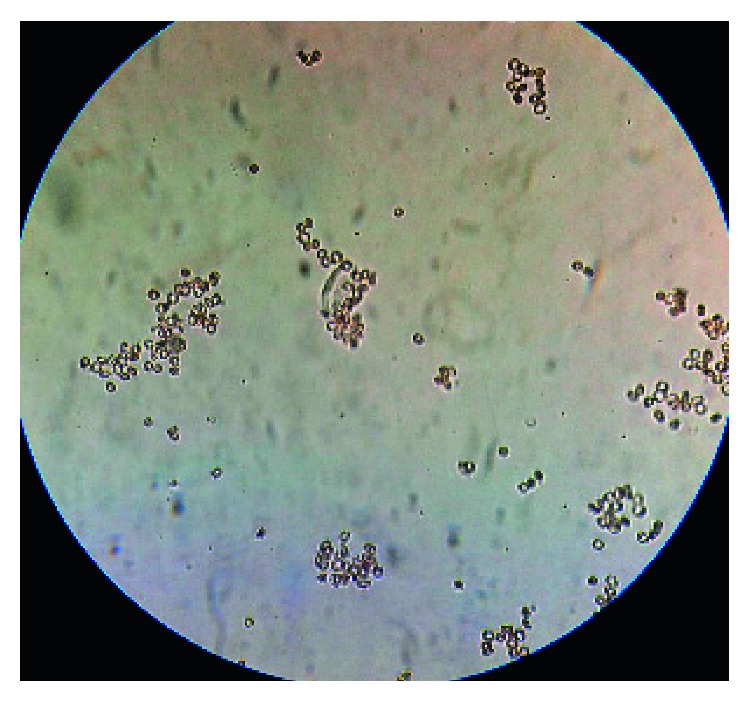
Clumping of red blood cell at 4°C in slide.

**Figure 7:**
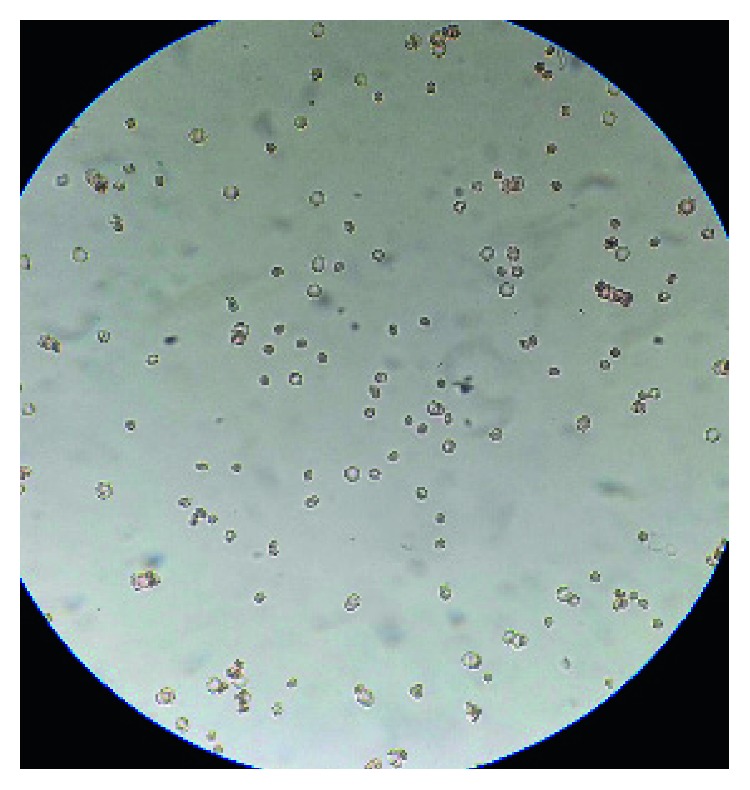
Disappearance of clumping while heating the slide at 37°C.

A 17-year-old young female from Kathmandu, Nepal, presented to the emergency department of the Institute of Medicine (IOM) with a generalized painful skin rash along with extensive blistering with mucosal involvement for one day (Figures 1–3). She had history of cough, sore throat, and fever few days prior to the appearance of rash for which she had taken azithromycin orally. On the 3^rd^ day of oral medication, she developed rash which was nonpruritic and painless. There was eruption of bumps starting from the trunk and spreading all over the body. Her eyelids and lip were swollen, and this was later associated with blistering and crusting. Around 20% skin detachment of body surface area was involved. The patient was clinically diagnosed as Stevens–Johnson syndrome-toxic epidermal necrolysis (SJS-TEN) overlap with tracheobronchitis. To find out the cause of SJS-TEN overlap, azithromycin was stopped. After 2 days of stopping azithromycin, her clinical symptoms did not improve, rather new lesions were seen. Then, the bedside cold agglutination test was done by cooling the blood taken in EDTA vial at 4°C. Before cooling, the blood formed smooth coating of the tube. After incubation at 4°C for 3 minutes, macroscopic hemagglutination was observed as cell clumping in thin film of blood that clinged to the tube (Figure 4). The clumping disappeared when the tube was warmed at 35.8°C and reappeared at 4°C (Figure 5). So, infective cause of SJS-TEN overlap was suspected, the most common cause being *Mycoplasma pneumoniae*. Blood was sent for the investigation of mycoplasma IgM and IgG antibodies. Also, serial dilutions of the patient's serum were mixed with an equal volume of 0.2% washed human O group erythrocytes, and clumping was observed till titer of 1 : 128 dilution after leaving at 4°C overnight (Figure 6). The clumping is dissociated at 37°C (Figure 7).

The complete blood count revealed total leukocyte count of 8000/*μ*l with lymphocytes of 60% with elevated ESR (70 mm/hour). Her liver enzymes and serum creatinine were normal. Test for syphilis (RPR and TPHA), human immunodeficiency virus (HIV), hepatitis B and C viruses, herpes simplex virus, Epstein–Barr virus, influenza A/B, and *Chlamydia pneumoniae* were negative.

**Figure 8:**
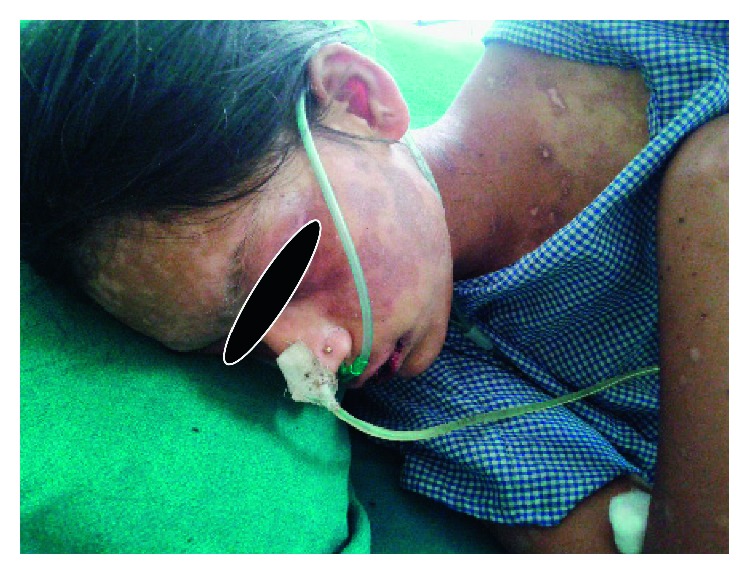
Patient improving on azithromycin and supportive treatment.

She was restarted with drug azithromycin and added hydrocortisone, paracetamol, betadine gargle, mupirocin, and ciprofloxacin ointment. Punch biopsy of her skin demonstrated subepidermal inflammation with necrotizing infundibular epithelium and necrotic keratinocytes consistent with SJS. Mycoplasma IgM antibody report was positive (2550 U/ml), which suggested the current infection and confirmed our diagnosis. The same treatment was continued and her clinical symptoms improved (Figure 8).

## 3. Discussion

SJS is a rare, emergency disorder of skin and mucous membranes that occurs secondary to use of certain drugs. The most common drugs causing SJS are anticonvulsants, sulfonamides, and oxicam nonsteroidal anti-inflammatory drugs [7]. SJS is classified as secondary to drugs when the patient has history of intake of offending drug within eight weeks before the onset of symptoms. SJS is classified as infectious if constitutional symptoms appear one week before the rash and the patient has positive serology [4]. Fever and viral prodrome-like symptoms are seen at an early stage, followed by skin and mucosal involvement. Mucosal lesion is more common and is seen in areas like oral, genital, and ocular region [2].

In the index case, initial presentation was fever, respiratory symptoms, and the involvement of oral mucosa. These features appeared after intake of antibiotic azithromycin. The first differential diagnosis was SJS-TEN secondary to the use of antibiotic. Cases of SJS/TEN secondary to the use of azithromycin have been reported earlier [8, 9]. SJS is thought to fall within a spectrum of diseases that affect the skin and mucous membranes, including erythema multiforme minor, erythema multiforme major (or SJS), and toxic epidermal necrolysis [6, 10]. Mycoplasma pneumonia can be associated with isolated mucous membrane disease or in combination with skin involvement [11]. Many authors believe that *M. pneumoniae*-associated mucositis with the minimal or absence of skin lesions is a separate entity from SJS, labelled as atypical SJS or *M. pneumoniae*-induced rash and mucositis [3, 12]. But in our case, both mucosal and skin involvement was seen. The term SJS is reserved for cases with <10% skin detachment of the body surface and TEN for those with >30%. Those with detachment of 10%–30% are termed as SJS-TEN overlap [1, 13]. More than 20% of skin detachment was seen in our case, so the diagnosis of SJS-TEN overlap was done.

Infectious cause of SJS-TEN overlap in our case was suspected when clumping occurred in cold agglutination test. Cold agglutination test has been used for bedside diagnosis of mycoplasma infection for very long time [14]. Furthermore, the increasing clinical severity after stopping azithromycin pointed to the infectious etiology of SJS-TEN overlap. Diagnosis of *Mycoplasma pneumoniae* infection is challenging due to the fastidious nature of the organism. Mycoplasmas are ubiquitous and are the smallest, free-living microorganisms. After an incubation period of 1 to 4 weeks, the infection typically presents with cough, pharyngitis, and rhinorrhea. Only 10% of patients develop pneumonia [4]. Extra pulmonary manifestations of MP infection are unusual and include SJS, arthritis, hemolytic anemia, and encephalitis [2]. Serology is the mainstay of laboratory diagnosis. When available, the polymerase chain reaction (PCR) is a rapid and helpful test, especially when combined with serology [5, 10].

In the resource-limiting country and hospitals where serology and PCR is not easily accessible, a simple bedside cold agglutination test can be done to increase the suspicion of infectious cause (most common *M. penumoniae*) of SJS-TEN overlap. The formation of cold agglutinins is the first humoral response to Mycoplasma [14]. Determination of these auto antibodies by cold agglutination test is fast and simple to perform. Since cold agglutinin is not a very reliable indicator, serology is the routine diagnostic modality for mycoplasma diagnosis. In our case, there was high titer (2550 U/ml) of mycoplasma IgM antibody. IgM antibodies appear during the first week of illness and reach maximum during third week [15].

The exact pathogenesis is unknown but immunological response to infectious agent causing generalized apoptosis of keratinocytes by T-lymphocytes and proteins like granulysin and Fas ligand has been postulated [6]. Supportive treatment was initiated with intravenous fluids and electrolytes in our patient. She was restarted with parenteral azithromycin and hydrocortisone was added intravenously. Local antibiotics like mupirocin and ciprofloxacin was applied for her skin lesion. Total parenteral nutrition was given which was subsequently stopped as she tolerated oral diet. She gradually made full recovery in two weeks' time. She was discharged on tapering dose of prednisolone and local antibiotics for remaining skin and mucosal lesion.

## 4. Conclusion

Mycoplasma pneumonia infection can cause SJS-TEN overlap with both skin and mucosal involvement. Besides considering the offending drugs, *Mycoplasma pneumoniae* infection should be considered in differential diagnosis of mucocutaneous lesion.
